# A chronic protocol of bilateral transcranial direct current stimulation over auditory cortex for tinnitus treatment: Dataset from a double-blinded randomized controlled trial

**DOI:** 10.12688/f1000research.14971.1

**Published:** 2018-06-12

**Authors:** Ali Yadollahpour, Miguel Mayo, Nader Saki, Samaneh Rashidi, Arash Bayat

**Affiliations:** 1 Department of Medical Physics, School of Medicine, Ahvaz Jundishapur University of Medical Sciences, Ahvaz, Iran; 2 Bioelectromagnetic Clinic, Imam Khomeini Hospital, Ahvaz Jundishapur University of Medical Sciences, Ahvaz, Iran; 3 Department of Otorhinolaryngology, A Coruña University Hospital Complex, A Coruña, Spain; 4 Hearing Research Center, Imam Khomeini Hospital, Ahvaz Jundishapur University of Medical Sciences, Ahvaz, Iran

**Keywords:** Transcranial direct current stimulation, repeated sessions, chronic protocol, Tinnitus, Auditory cortex, Tolerability, Adverse effects

## Abstract

Preliminary studies have demonstrated the therapeutic potential of transcranial direct current stimulation (tDCS) for chronic tinnitus. However, the findings are controversial and most of the studies investigated effects of a single session of tDCS and short after-effects, ranging from hours to days. To our knowledge, there is no published study investigating the effects of a chronic protocol of bilateral tDCS over auditory cortex (AC) with one month follow-up in a double blinded randomized clinical trial. This dataset presents the results of a double-blinded placebo controlled trial investigating the effects of chronic protocol (10 sessions) of tDCS over AC with 1 month follow-up. The data of the two groups, real tDCS (n=25) and sham tDCS (n=15), are reported. The dataset includes three main data groups: patient- and tinnitus-specific data, data of the primary and secondary outcomes, and data on the adverse effects of and tolerability to tDCS. The first group includes demographic information, audiometric assessments, and tinnitus-specific characteristics. The second group includes tinnitus handicap inventory (THI) scores, tinnitus loudness, and tinnitus related distress based on 0-10 numerical visual analogue scale (VAS) scores. The values of the primary and secondary outcomes for pre-intervention and at different time points following interventions are presented. THI scores pre-intervention and immediately post-intervention and at 1 month follow-up; the scores of tinnitus loudness and distress scores for pre-intervention, and immediately, 1 hour, 1 week, and at 1 month after the last stimulation session are presented. Moreover, the adverse effects of and tolerability to the tDCS were assessed using a customized questionnaire after the last tDCS session. This dataset can be used alone or in combination with other datasets using advanced statistical analyses and modeling to investigate the treatment efficacy of tDCS in chronic intractable tinnitus.

## Introduction

Tinnitus is a phantom auditory perception in the absence of external sound that affects 10–15% of the world adult population, present in different forms, including buzzing, hissing, pulsatile, ringing and pulsatile tone ^1, 2^. Neuroimaging studies have shown that tinnitus is a complex disorder involving a large network consisting of multiple overlapping brain networks including primary and secondary auditory cortices, as well as specific non-auditory areas and limbic processes ^3, 4^. Several pharmaceutics agents are used for tinnitus treatment; however, a large portion of the patients are resistant to the treatment, which usually induce severe comorbidities, such as anxiety, sleep disturbances and depression ^2^. There is currently no definitive medication-based treatment for tinnitus ^1– 3^. Different research groups have proposed and developed several non-pharmacological interventions, including cognitive behavioral therapies, hearing aids, neurofeedback, and noise-masking techniques ^5– 7^. Despite the development of different non-pharmaceutical techniques including cognitive behavioral therapies ^8^, noise-masking modalities ^9^, and neurofeedback ^10^, the efficacies of these treatments for tinnitus are limited.

Transcranial direct current stimulation (tDCS) has potential therapeutic efficacy for different neuropsychiatric disorders ^5– 7, 11, 12^ and also that capability of enhancement of cognitive functions in healthy individuals ^13^. Single and repeated-session protocols of tDCS over the auditory cortex (AC) have shown promising outcomes ^14, 15^; however, the findings are controversial and most of the studies that have been conducted investigated the effects of single-session protocols of tDCS and short after-effects, ranging from hours to some days ^15, 16^. To achieve an effective tDCS protocol, different clinical studies with large sample sizes and robust designs should be conducted to identify the effective electrode montage and stimulation parameters. In addition, chronic protocols with long enough follow-up assessments should also be considered. For long-term tDCS protocols, assessing the possible adverse effects is necessary. In this regard, we have designed a comprehensive project to assess the efficacy of different protocols of tDCS with different electrode montages over different sites of the brain for intractable tinnitus ^17^. Here, we report the dataset of a clinical trial designed as a double-blinded randomized placebo-controlled trial to investigate the effects of repeated sessions of tDCS on tinnitus symptoms. To the best of our knowledge, this is the first randomized clinical trial to investigate the effects of repeated sessions of tDCS on intractable chronic tinnitus symptoms and comorbid depression and anxiety with a 1-month follow-up. The main feature of these data is that the patients, the researcher who evaluated the outcomes, and the researcher who performed the statistical analyses were blinded to the study.

## Methods

### Participants and dataset schema

This dataset presents the results of a double blinded randomized placebo controlled clinical trial investigated the effects of chronic protocol of bilateral tDCS over AC in intractable chronic tinnitus (n=40) ^18^. It should be noted that the main study protocol was designed with three arms (anode, cathode, and placebo, each with 30 patients) ^18^. However, due to several reasons we decided to conduct and report the results of the two arms of the protocol as a separate study. The two arms were anode (real group) and placebo groups. The remaining arms of the original study are under recruitment phase and will be conducted with two arms of cathode (n=30) and placebo (n=30). The main reason was difficulty in recruiting enough patients who meet the inclusion/exclusion criteria so that we could not conduct the whole study as a single trial within a season, and the duration of the study took a long time. In addition, we observed a significant different therapeutic outcomes between the real (anode arm) and placebo groups and decided to report the two arms as a separate study. The primary outcomes of this study are expected to be reported within approximately 4 months.

The data consist of three main groups: the first group is demographic information, tinnitus characteristics and audiometric assessments of the patients; the second group are data of the primary and secondary outcomes at pre- and post-intervention; and the third group are data concerning the adverse effects and tolerability of tDCS. Table 1 presents the demographic information, tinnitus characteristics (including tinnitus quality, laterality and duration), audiometric assessments, and the primary and secondary outcomes for pre- and post-intervention for the participants in the real tDCS. The corresponding data for the sham tDCS group are presented in Table 2. Table 3 and Table 4, respectively, present the adverse effects and tolerability of tDCS for real and sham tDCS groups using a customized questionnaire ( Supplementary File 1).

**Table 1.** Demographic information, tinnitus characteristics, audiometric assessments, and primary and secondary outcomes of the participants in the real tDCS.

Patient code	Age, y	Sex	Quality ^a^	Laterality ^b^	Duration(y)	THI			Loudness					Distress					
						pre	Post	Post-1m	pre	Post-i	Post-1h	Post-1w	Post-1m	pre	Post-i	Post-1h	Post-1w	Post-1m	hearing loss right/ left ^c^
1	55	F	HPW	L	10	81	75	80	8	8	8	8	8	8	7	7	7	8	M/P
2	43	F	P	R > L	4	67	44	46	7	5	5	5	6	8	5	5	6	7	N/L
3	47	F	R + T	L	11	70	65	60	8	8	8	8	8	8	8	8	8	8	P/L
4	63	F	HPW + TH	R > L	3	75	35	36	8	5	5	5	6	8	6	6	6	7	M/L
5	48	F	R	R	8	91	40	44	7	4	4	5	6	8	5	5	6	6	N/N
6	42	F	R	R = L	10	70	42	47	7	3	3	4	6	8	6	6	7	7	N/N
7	38	F	C	R=L	3	75	35	39	7	4	4	4	6	8	5	5	5	6	N/N
8	54	F	R + H	R	5	56	35	36	7	5	5	5	7	8	5	5	5	7	N/L
9	55	F	H + B	R < L	15	72	65	71	8	8	8	8	8	7	6	6	7	7	M/L
10	33	F	R	R > L	7	54	28	30	7	3	3	3	5	6	4	4	4	5	N/N
11	48	F	P	L	12	67	31	40	9	6	5	5	8	8	4	4	4	6	L/L
12	43	F	HPW	R	15	86	50	70	8	6	6	6	8	8	6	6	6	8	M/M
13	42	F	R	R>L	3	77	34	38	6	4	4	5	5	7	5	5	5	6	L/N
14	47	F	H + B	R	2	83	45	49	7	5	5	5	6	8	6	6	6	7	M/L
15	57	M	B	L > R	12	66	60	68	6	6	6	6	7	7	7	7	7	7	L/M
16	47	M	C	R	5	89	43	49	7	4	4	5	6	7	5	5	6	6	L/N
17	48	M	C	L > R	5	60	36	41	6	5	5	5	6	8	6	6	7	7	L/N
18	38	M	R	R > L	4	56	30	28	7	5	5	5	5	7	5	5	5	6	N/N
19	48	M	H	R	5	57	32	38	8	5	5	5	6	8	6	6	6	7	N/N
20	52	M	C	R < L	8	75	50	57	7	6	6	6	7	8	7	7	7	8	N/N
21	62	M	P	R	6	73	40	43	7	4	4	4	5	8	6	6	6	7	N/L
22	45	M	R	R>L	6	66	37	39	7	5	5	5	6	7	4	4	4	5	L/L
23	50	M	R	L	7	59	55	60	8	9	9	8	8	8	8	8	8	8	P/L
24	36	M	HPW	R	6	77	68	60	9	9	9	9	9	8	8	8	8	9	P/M
25	47	M	R	R< L	15	80	85	73	8	8	8	8	8	8	8	8	8	8	M/N

THI, Tinnitus Handicap Inventory; pre, pre-intervention; post, post-intervention; post-i, immediately after intervention; post-1h, at 1 hour post-intervention; post-1w, at 1 week post-intervention; post-1m, at 1 month post-intervention. Tinnitus loudness and distress ranged 0–10, where 0 indicates the lowest level and 10 indicates the highest tolerable level. ^a^Tinnitus quality codes: R, ringing; B, buzzing; H, hissing; HU, humming; T, ticking; HPW, high-pitched whistling; TH, thumping; C, cicadas; P, pulsating. ^b^Tinnitus side: L, left; R, right, R = L, bilateral with no lateralization; R>L, bilateral lateralizing more to the right side; L>R, bilateral lateralizing more to the left side. ^c^Class of hearing loss: N, normal hearing threshold (<20 dB); L, mild hearing loss (20–40 dB); M, moderate hearing loss (41–70 dB); S, severe hearing loss (70–90 db); P, profound hearing loss (<90 db).

**Table 2.** Demographic information, tinnitus characteristics, audiometric assessments, and primary and secondary outcomes of the participants in the sham transcranial direct current stimulation.

Patient code	Age, y	Sex	Quality ^a^	Laterality ^b^	Duration(y)	THI			Loudness					Distress					
						pre	Post-i	Post-1m	pre	Post-i	Post-1h	Post-1w	Post-1m	pre	Post-i	Post-1h	Post-1w	Post-1m	hearing loss right/ left ^c^
1	55	F	HPW	L	10	81	75	77	8	8	8	8	8	8	7	8	8	8	M/P
2	43	F	P	R	4	67	60	62	7	6	7	7	7	8	8	8	8	8	N/L
3	47	F	R + T	R	8	70	65	67	8	8	8	8	8	8	8	8	8	8	P/L
4	63	F	HPW + TH	R>L	7	75	70	72	8	8	8	8	8	8	8	8	8	8	L/M
5	48	F	R	R<L	4	91	87	88	7	6	7	7	8	8	8	8	8	9	N/L
6	42	F	R	L	9	70	69	67	9	9	9	9	9	8	8	8	7	8	N/N
7	38	F	C	L	4	75	49	70	7	6	6	7	7	8	7	8	8	8	N/N
8	54	F	R + H	R=L	6	56	50	51	7	7	7	7	7	8	8	8	8	8	L/N
9	55	M	H + B	L	12	72	65	62	8	8	8	8	8	7	6	6	7	7	M/L
10	33	M	R	R	4	54	38	42	7	5	5	7	7	6	4	5	7	7	N/N
11	48	M	P	R>L	15	67	58	55	9	9	9	9	9	8	8	8	8	8	L/L
12	43	M	HPW	R	12	86	88	80	8	7	8	8	8	8	7	8	8	8	M/M
13	42	M	R	R>L	5	77	75	63	6	4	4	5	6	7	5	5	5	7	L/N
14	47	M	H + B	L	4	83	84	80	7	5	7	7		8	7	8	7	8	M/L
15	57	M	B	R<L	10	70	68	65	6	6	6	6		7	7	7	7	7	L/M

THI, Tinnitus Handicap Inventory; pre, pre-intervention; post, post-intervention; post-i, immediately after intervention; post-1h, at 1 hour post-intervention; post-1w, at 1 week post-intervention; post-1m, at 1 month post-intervention. Tinnitus loudness and distress ranged 0–10, where 0 indicates the lowest level and 10 indicates the highest tolerable level. ^a^Tinnitus quality codes: R, ringing; B, buzzing; H, hissing; HU, humming; T, ticking; HPW, high-pitched whistling; TH, thumping; C, cicadas; P, pulsating. ^b^Tinnitus side: L, left; R, right, R = L, bilateral with no lateralization; R>L, bilateral lateralizing more to the right side; L>R, bilateral lateralizing more to the left side. ^c^Class of hearing loss: N, normal hearing threshold (<20 dB); L, mild hearing loss (20–40 dB); M, moderate hearing loss (41–70 dB); S, severe hearing loss (70–90 db); P, profound hearing loss (>90 db).

**Table 3.** Adverse effects (AEs) in the real transcranial direct current stimulation group (n=25).

Effect	Total, n (%)	Mild, n (%)	Moderate, n (%)	Significant, n (%)	Very high, n (%)	Site of AE ^a^
Itching	19 (76%)	14 (73.7%)	4 (21.1%)	1 (5.2%)	0	C
Tingling	17 (68%)	13 (76.5%)	3 (17.6%)	1 (5.9%)	0	C
Scalp Pain	2 (8%)	2	0	0	0	A
Burning	2 (8%)	1 (50%)	1 (50%)	0	0	C
Vertigo	0	0	0	0	0	NA
Pinching	4 (16%)	2 (50%)	2 (50%)	0	0	C
Metallic/iron taste	0	0	0	0	0	NA
Fatigue	2 (8%)	2 (100%)	NA	NA	NA	NA
hypomania	0	0	0	0	0	NA
Heat	0	0	0	0	0	NA
headache	4 (16%)	3 (75%)	1 (25%)	0	0	NA
Skin irritation	10 (40%)	8 (80%)	2 (20%)	0	0	A
Dizziness	0	0	0	0	0	NA
Discomfort	5 (20%)	3 (60%)	2 (40%)	0	0	NA
Tolerability						
Very high	23 (92%)	NA	NA	NA	NA	NA
Moderate	4 (16%)	NA	NA	NA	NA	NA
Mild	2 (8%)	NA	NA	NA	NA	NA
None	0	NA	NA	NA	NA	NA

NA, not applicable. ^a^C, under cathode; A, under anode. Very high tolerability indicates the subject could easily tolerate the tDCS sessions.

**Table 4.** Adverse effects (AEs) of the sham transcranial direct current stimulation (n=15).

Effects	Total, n (%)	Mild, n (%)	Moderate, n (%)	Significant, n (%)	Very high, n (%)	Site of AE ^a^
Itching	8 (53.3%)	7 (87.5%)	1 (12.5%)	0	0	C
Tingling	7 (46.6%)	6 (85.7%)	1 (14.3%)	0	0	C
Scalp Pain	1 (6.6%)	1(100%)	0	0	0	A
Burning	2 (13.3%)	1 (50%)	1 (50%)	0	0	C
Vertigo	0	0	0	0	0	NA
Pinching	3 (20%)	2 (75%)	1 (25%)	0	0	C
Metallic/iron taste	0	0	0	0	0	NA
Fatigue	1 (66.6%)	1 (100%)	0	0	0	NA
hypomania	0	0	0	0	0	NA
Heat	0	0	0	0	0	NA
headache	2 (13.3%)	2 (100%)	0	0	0	NA
Skin irritation	5 (33.3%)	5 (100%)	0	0	0	A
Dizziness	0	0	0	0	0	NA
Discomfort	5 (33.3%)	4 (80%)	1 (20%)	0	0	NA
Tolerability						
Very high	14 (93.3%)	NA	NA	NA	NA	NA
Moderate	2 (13.3%)	NA	NA	NA	NA	NA
Mild	1 (6.6%)	NA	NA	NA	NA	NA
None	0	NA	NA	NA	NA	NA

^a^C:under cathode, A:under anode, C-A: between cathode and anode; very high tolerability indicates the subject could easily tolerate the tDCS sessions.

### Study design

All of the experimental procedures of the study were approved by local ethical committee of Ahvaz Jundishapur University of Medical Sciences (AJUMS), Ahvaz, Iran (registration code: IR.AJUMS.REC.1394.639), and were completely in accordance with the ethical standards and regulations of human studies of the Helsinki declaration (2014). After the enrolment, objectives, possible benefits, and side effects of the study were clearly explained to the patients and all patients filled and signed a written consent form for participation in the study.

**Figure 1.**
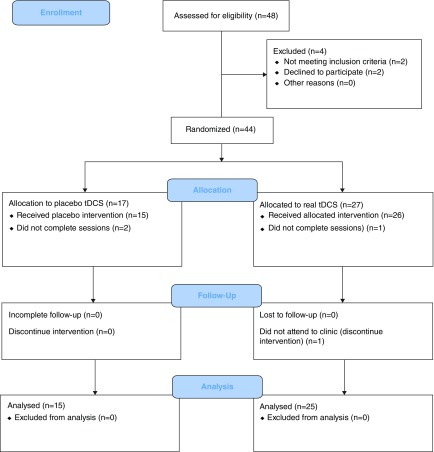
CONSORT diagram of the study.

Patients with intractable chronic tinnitus (n=40) were randomly assigned into real tDCS (n=25; female, 14; male, 11; age, 47.52 ± 7.51 years; disease duration, 7.48 ± 3.99 years) and placebo tDCS (n=15; female, 8; male, 7; age, 47.67 ± 7.96 years; disease duration, 7.60 ± 3.60 years) in a parallel allocation and a double-blinded randomized controlled clinical trial ( Figure 1). Due to the difficulty in the patient recruitment and high number of the patients who did not complete the treatment we reduced the size in the anode and placebo group (n=25 vs n=30 for the anode group and n=15 vs n=30 for the placebo group). The change in the groups’ size was approved by the local ethics committee of Ahvaz Jundishapur University of Medical Sciences, Ahvaz, Iran (registration code: IR.AJUMS.REC.1394.639). The treatment consisted of daily sessions of 20 minutes of 2 mA current for 5 consecutive days per week and 2 consecutive weeks with 35 cm ^2^ electrodes. Both groups were matched in age, gender, ethnicity, and audiometric main characteristics. The patients, the researcher who evaluated the outcomes during the experiments and at the follow-up period, and the researchers who performed data analyses were blinded to the type of protocol. The experimental procedures of the present study, including tDCS sessions and outcomes evaluations, were performed at the Bioelectromagnetic Clinic in Ahvaz Imam Hospital, an affiliated Hospital to AJUMS, Iran. The study was registered as a clinical trial in the Iranian Registry of Clinical Trial ( IRCT2016110124635N6), which is the registration for the original clinical trial design ^18^.

### TDCS intervention

The direct current was applied through a saline-soaked pair of carbon electrodes (35 cm ^2^) and delivered by a tDCS device (OASIS Pro ^TM^; Mind Alive, Inc., Edmonton, Alberta, Canada). The tDCS protocol consisted of 2 mA current, daily for 20 min, over 5 consecutive days per week for 2 consecutive weeks (total, 10 sessions). The anode was placed over left AC (halfway T3 - F7) and cathode over right AC (halfway T4 - F8) with 35 cm ^2^ electrodes. In the placebo tDCS, the electrode montage was the same with real tDCS except that the device was turned off after 30 s after the start of session without the patient knowing it ^19^. During treatment, the patients were asked to remove all metal-based jewelry from the head and neck.

### Clinical evaluation

Before the start of the first session of tDCS, intervention, all patients underwent complete audiometric and neurological assessments by expert specialties and the data were recorded along with the demographic data ( Table 1 and Table 2). The recorded variables consisted of the tinnitus quality, lateralization, duration, and class of hearing loss in both ears ( Table 1 and Table 2). The lateralization or tinnitus side is the dominant side of the head where the patient experiences the tinnitus and was classified as left (L), right (R), bilateral with no lateralization (R = L), bilateral lateralizing more to the right side (R>L), bilateral lateralizing more to the left side (L>R). In addition, hearing assessments were conducted in an acoustically isolated chamber with pure-tone audiometry using an AC 40 dual-channel Audiometer (Intracoustics Co., Denmark). The hearing thresholds were recorded over the frequency ranges of 250 to 8000 Hz for air conduction and 500 to 4000 Hz for bone conduction pathways, according to the modified Hughson–Westlake Method proposed by ANSI 1997 ^20^. Pure-tone audiometry was considered normal whenever the hearing thresholds at all frequencies were below 20 decibels hearing level (dBHL). The class of hearing loss in both ears was classified as normal hearing threshold (<20 dB), mild hearing loss (20–40 dB), moderate hearing loss (41–70 dB), severe hearing loss (70–90 db), and profound hearing loss (>90 db). The hearing class was determined as the average of threshold in 250, 1000, 2000, and 4000 Hz ^20^. The THI scores were assessed at pre-intervention, immediately after intervention, and at 1 month after the last stimulation. The tinnitus loudness and distress were recorded using a numeral 0–10 VAS rating scale before intervention, and immediately, 1 hour, 1 week, and 1 month after last stimulation.

### Adverse effects and tolerability

Previous studies have demonstrated that tDCS is relatively safe with no serious side effects ^11, 15, 19^. It should be mentioned that most of the conducted studies on the safety profile of tDCS assessed during single-session tDCS with different current intensities. For this study, considering the chronic protocol of tDCS, the adverse effects of and tolerability to the chronic protocol of tDCS were assessed using a customized questionnaire ( Supplementary File 1). We used a five-point Likert-type scale for each adverse effect and the tolerability. In addition, the site (under cathode, under anode, other (mention) and time of sensation (beginning, middle, and end of session), and duration of the sensation (very short, some minutes, throughout, and after termination of session) end for each session were recorded. The questionnaire was filled before the start of the tDCS intervention and after the last session.

### Quality assurance

The measured data were recorded in customized designed forms which were available in print. All the collected data were entered into the specific forms in the Excel (Microsoft Office, 2010). The data entry was double-checked by two independent researchers. After validation of the data by a third researcher, the data were checked for wrong and out-of-range values and any dispute was resolved referring to the print version of the dataset. For the missing data, the missing data point was imputed using linear interpolation to reach the averaged values of the sequential closest values. After the validation, the data were sorted and then entered into statistical package of SPSS (Version 20, Windows) for analyses.

## Utility and discussion

This dataset has two main advantages over similar studies so far conducted. First, tinnitus-specific features as well as audiometric measures, including the laterality and quality of tinnitus, pure tone auditory threshold and class of hearing impairments for both ears, are assessed and recorded for all patients. This feature allows researchers to investigate the possible correlations between the response and/or non-response to tDCS and each of these variables, along with gender and history of tinnitus. However, to find reliable correlations, it is necessary to increase the volume of such datasets, and the similar data from other clinical trials can help to build a comprehensive dataset of tinnitus-specific features. The second feature is that this dataset presents the effects of relatively long-term tDCS exposure (10 sessions, one session daily) on tinnitus symptoms; the primary and secondary outcomes were measured at several time points following intervention for 1 month. The outcomes were assessed at different time points covering the short and long term after effects. A 1-month follow-up is relatively long compared to the similar studies ^14– 16^ conducted so far; however; it is necessary to increase the follow-up assessments further to several months. Such datasets can be used alone or in combination with other datasets, using advanced statistical analyses and modeling to identify the influencing parameters of tDCS as well as patient-specific features correlating with the therapeutic outcomes of the tDCS.

## Conclusion

This dataset presents the effects of tDCS in tinnitus symptoms in a double-blinded randomized placebo-controlled trial with a 1-month follow-up. Considering the long-term tDCS exposure, the AEs and tolerability to tDCS were also presented. One of the limitations of this study was relatively low sample size, and the imbalance in sample size between the real and sham tDCS groups. For the latter limitation, which could result in overestimation of the effect size in the real tDCS groups, the authors could conduct bootstrap analyses to determine whether overestimation or underestimation occurred in each group. In line with this trial, our group is conducting a series of clinical trials to reach an effective tDCS treatment for tinnitus. Adding the dataset of other trials to this data will allow researchers to quantitatively evaluate the factors influencing the treatment efficacy and to build different treatment models for tinnitus based on the tinnitus-specific features.

## Data availability

The datasets of this study are freely available in the Mendeley repository under a CC BY 4.0 license, DOI: https://doi.org/10.17632/8d8wrk62vy.1 ^21^.

## Abbreviations

AC: auditory cortex; tDCS: transcranial direct current stimulation; THI: tinnitus handicap inventory; BDI-II: Beck Depression inventory; BAI: Beck Anxiety Inventory; dBHL: decibels hearing level.

## Supplementary material

**Supplementary File 1. Adverse effects and tolerability questionnaire.**
