# Supplementary material for: A chronic protocol of bilateral transcranial direct current stimulation over auditory cortex for tinnitus treatment: Dataset from a double-blinded randomized controlled trial
Source: F1000Res. 2018 Jun 12;7:733. [Version 1] doi: 10.12688/f1000research.14971.1 (PMC6178906; doi:10.12688/f1000research.14971.1)
Supplement: Supplementary file 1 [file f1000research-7-16299-s0000.tgz › 09cd3465-e166-4bea-a968-664bef6fa182.docx]

**Subject code:** _______________________________________________ **Date**: **/** **/** |

**Experiment:** _____________________________________________________________________

This questionnaire aims to investigate adverse effects and any perception you experience during the tDCS session and after the session. Please answer the following items on the different perceptions or sensations on the intensity of your experience according to the following criteria:

- ***Not at all* = I did not feel the described sensation (0)**
- ***Mild* = I mildly felt the described sensation (1)**
- ***Moderate* = I felt the described sensation (2)**
- ***Considerable* = I felt the described sensation to a considerable degree (3)**
- ***Strong* = I strongly felt the described sensation (4)**

| Patient code:  Session No.# | none | mild | moderate | significant | very high | Site of sensation | | | Time of sensation  (each session) | | | Duration | | | |
| --- | --- | --- | --- | --- | --- | --- | --- | --- | --- | --- | --- | --- | --- | --- | --- |
|  |  |  |  |  |  | Under Cathode | Under anode | Other regions (where) | Beginning | Middle | End | Very short | Some minutes | Throughout session | After end |
| Itching |  |  |  |  |  |  |  |  |  |  |  |  |  |  |  |
| Tingling |  |  |  |  |  |  |  |  |  |  |  |  |  |  |  |
| Scalp Pain |  |  |  |  |  |  |  |  |  |  |  |  |  |  |  |
| Burning |  |  |  |  |  |  |  |  |  |  |  |  |  |  |  |
| Vertigo |  |  |  |  |  |  |  |  |  |  |  |  |  |  |  |
| Pinching |  |  |  |  |  |  |  |  |  |  |  |  |  |  |  |
| Metallic/Iron taste |  |  |  |  |  |  |  |  |  |  |  |  |  |  |  |
| Fatigue |  |  |  |  |  |  |  |  |  |  |  |  |  |  |  |
| hypomania |  |  |  |  |  |  |  |  |  |  |  |  |  |  |  |
| Heat |  |  |  |  |  |  |  |  |  |  |  |  |  |  |  |
| headache |  |  |  |  |  |  |  |  |  |  |  |  |  |  |  |
| Skin irritation |  |  |  |  |  |  |  |  |  |  |  |  |  |  |  |
| dizziness |  |  |  |  |  |  |  |  |  |  |  |  |  |  |  |
| Discomfort |  |  |  |  |  |  |  |  |  |  |  |  |  |  |  |
| tolerability |  |  |  |  |  |  |  |  |  |  |  |  |  |  |  |

***Blinding assessment****: please ask the patients at the end of intervention*

Do you believe that you received a real or placebo stimulation?

real □ placebo □ I don’t know □

Assessment (filled by researcher): correct □ wrong □

**To researcher/clinician:**

**Please fill the questionnaire after each session of tDCS.**

Please report any adverse event/problem that occurred and rate the event/problem on a scale from 1 to 4 as previously described.

________________________________________________________________________________________________________________________________________________________________________________________________________________________________________________________________________________________________________________________________
