# Supplementary material for: A chronic protocol of bilateral transcranial direct current stimulation over auditory cortex for tinnitus treatment: Dataset from a double-blinded randomized controlled trial
Source: F1000Res. 2018 Jun 12;7:733. [Version 1] doi: 10.12688/f1000research.14971.1 (PMC6178906; doi:10.12688/f1000research.14971.1)
Supplement: Peer review history

# Peer review: Referee response for version 1

**DOI:** 10.5256/f1000research.16299.r38489

**Published:** 2018-10-08

**Authors:** Saeid Mahmoudian

**Affiliations:** 1 ENT and Head & Neck Research Center and Department, Iran University of Medical Sciences (IUMS) , Tehran, Iran

The authors present a well-protocol using bilateral transcranial direct current stimulation over auditory cortex for tinnitus treatment. 

 Tinnitus is quite common, yet not well understood. Understanding the etiology and neural basis of tinnitus would help in developing treatment: both alleviation and even remediation. In addition brain research on tinnitus can also reveal important information on the auditory system and its principles in general. As in many neurological conditions, the challenge in studying tinnitus lies in its heterogeneity and the comorbidity with other conditions affecting auditory processing. When regarding tinnitus the hearing loss is perhaps the most obvious mixing factor. In my opinion, this protocol provides a fairly good starting point towards investigating the effects of a chronic protocol of bilateral tDCS over auditory cortex (AC) with one-month follow-up in a double-blinded randomized clinical trial. The study is reasonably well planned and conducted. However, there are some concerns about the efficacy and safety of tDCS and finding a specific electrode location on the patient's scalp in order to achieve optimal electrode positioning.

I have read this submission. I believe that I have an appropriate level of expertise to confirm that it is of an acceptable scientific standard.

## References

[1] MohsenSPourbakhtAFarhadiMMahmoudianS: The efficacy and safety of multiple sessions of multisite transcranial random noise stimulation in treating chronic tinnitus. *Brazilian Journal of Otorhinolaryngology*.2018; 10.1016/j.bjorl.2018.05.010 10.1016/j.bjorl.2018.05.010 PMC944304830528654

[2] MohsenSMahmoudianSTalebianSPourbakhtA: Prefrontal and auditory tRNS in sequence for treating chronic tinnitus: a modified multisite protocol. *Brain Stimulation*.2018;11(5) : 10.1016/j.brs.2018.04.018 1177-1179 10.1016/j.brs.2018.04.018 29730252

# Peer review: Referee response for version 1

**DOI:** 10.5256/f1000research.16299.r34973

**Published:** 2018-07-03

**Authors:** Myles Jones

**Affiliations:** 1 Department of Psychology, University of Sheffield, Sheffield, UK

The study is well designed, double-blinded and randomized. Despite some non-completions the study is still reasonably ‘well-powered’, in that the n is reasonable compared to that typically reported in the literature. There is a clear flow diagram explaining how subjects were assigned to conditions. There is a wide range of outcome measures including those of auditory function, perception of tinnitus and well-being. There are several outcome time points which is also a strength of the design.

 The introduction may have liked to have provided a rationale for only investigating cathodal rather than anodal TDCS. Presumably the putative inhibitory actions of cathodal TDCS (rather than the excitatory actions of anodal TDCS) would be expected to reduce tinnitus symptoms.

I have read this submission. I believe that I have an appropriate level of expertise to confirm that it is of an acceptable scientific standard.
